# Diagnosis of skin cancer by correlation and complexity analyses of damaged DNA

**DOI:** 10.18632/oncotarget.6003

**Published:** 2015-10-16

**Authors:** Hamidreza Namazi, Vladimir V. Kulish, Fatemeh Delaviz, Ali Delaviz

**Affiliations:** ^1^ School of Mechanical and Aerospace Engineering, Nanyang Technological University, Singapore; ^2^ Faculty of Chemical Engineering, Islamic Azad University (Fars Science and Research Branch), Shiraz, Iran; ^3^ Faculty of Mechanical Engineering, Iran University of Science and Technology, Tehran, Iran

**Keywords:** skin cancer, DNA walk, fractal dimension, the Hurst exponent, damaged DNA

## Abstract

Skin cancer is a common, low-grade cancerous (malignant) growth of the skin. It starts from cells that begin as normal skin cells and transform into those with the potential to reproduce in an out-of-control manner. Cancer develops when DNA, the molecule found in cells that encodes genetic information, becomes damaged and the body cannot repair the damage. A DNA walk of a genome represents how the frequency of each nucleotide of a pairing nucleotide couple changes locally. In this research in order to diagnose the skin cancer, first DNA walk plots of genomes of patients with skin cancer were generated. Then, the data so obtained was checked for complexity by computing the fractal dimension. Furthermore, the Hurst exponent has been employed in order to study the correlation of damaged DNA. By analysing different samples it has been found that the damaged DNA sequences are exhibiting higher degree of complexity and less correlation compared to normal DNA sequences. This investigation confirms that this method can be used for diagnosis of skin cancer. The method discussed in this research is useful not only for diagnosis of skin cancer but can be applied for diagnosis and growth analysis of different types of cancers.

## INTRODUCTION

Skin cancer is a type of cancer that arises from skin. It is the most common form of cancer, globally accounting for at least 40% of cases [1]. It is especially common among people with light skin. Skin cancer is due to the development of abnormal cells that have the ability to invade or spread to other parts of the body. There are three main types: basal cell cancer (BCC), squamous cell cancer (SCC) and melanoma. The most common type is non-melanoma skin cancer, which occurs in at least 2–3 million people per year. Of non-melanoma skin cancers, about 80% are basal cell cancers and 20% squamous cell cancers. Basal cell and squamous cell cancers rarely result in death [2].

One of the main reasons that skin cancer develops is because the DNA is damaged. DNA is the master molecule that controls and directs every cell in the body. Damage to DNA is one of the ways that cells lose control of growth and become cancerous. DNA mutations can also be inherited.

During years few methods have been investigated in order to diagnose the skin cancer. These methods are mainly categorized in two types which are skin biopsy, and image analysis [3]. In case of skin biopsy doctor takes a sample of skin from the suspicious area to be looked at under a microscope. Different methods can be used for a skin biopsy. The doctor will choose one based on the size of the affected area, where it is on the patient's body, and other factors.

In case of image analysis of damaged skin using computers, special features, like particular colours, colour variation and texture are analysed to search for the sign of cancer. In fact, image analysis methods are based on mathematics. For instance, Segura et al. performed a systemic analysis of melanocytic and non-melanocytic skin tumors, using dermatoscopy, RCM, and histopathology to develop a two-step method for melanoma diagnosis based on RCM features for use as an adjuvant to dermatoscopy [4]. In another extensive work Stoecker et al. analyzed asymmetry, as a critical feature in the diagnosis of malignant melanoma, using a new algorithm to find a major axis of asymmetry and calculate the degree of asymmetry of the tumor outline [5]. See also [6–9].

Thermal image analysis of damaged skin can be stated as a special type of image analysis. In this category limited works have been reported in literatures. Flores-Sahagun et al. proposed a structured methodology for analysis and diagnosis of basal cell carcinoma (BCC) via infrared imaging temperature measurements. They concluded that their conjugated gradients method was efficient to identify lesioned tissue (which was associated to basal cell carcinoma through a clinical exam together with skin biopsies) in all patients studied even with the use of a camera of low optical resolution (160 × 120 pixels) and thermal resolution of 0.1°C [10]. Poljak-Blazi et al. also employed infrared thermal imaging for evaluation of the tumour development and discrimination of cancer from inflammation and haematoma [11]. See also [12].

Fractals are defined to be scale-invariant (self-similar or self-affine) geometric objects. A geometric object is called self-similar if it may be written as a union of rescaled copies of itself, with the rescaling isotropic or uniform in all directions. Regular fractals display exact self-similarity. Random fractals display a weaker, statistical version of self-similarity or, more generally, self-affinity. Although virtually all natural fractals are random, the concept of self-similarity is best first explored through the study of regular fractals.

The class of regular fractals includes many familiar simple objects such as line intervals, solid squares, and solid cubes, and also many irregular objects. The scaling rules are characterized by “scaling exponents” (dimension). “Simple” regular fractals have integer scaling dimensions. Complex self-similar objects have non-integer dimension. Therefore, it is completely incorrect to define fractals as geometric objects having “fractional” (non-integer) dimension. Fractals may be defined as geometric objects whose scaling exponent (dimension) satisfies the Szpilrajn inequality:

$$ℵ\geq D_{T}$$ (1)

where ℵ is the scaling exponent (dimension) of the object and *D_T_* is its topological dimension, i.e., Euclidean dimension of units from which the fractal object is built. For example, in case of Brownian motion: the path of a particle, a line of dimension one, traveling for a long time over a plane region, eventually covers the entire plane, an entity of dimension two [13].

A multi-fractal system is a generalization of a fractal system in which a single exponent (the fractal dimension) is not enough to describe its dynamics; instead, a continuous spectrum of exponents (the so-called singularity spectrum) is needed.

There are limited works which have employed fractal dimension under image analysis techniques in order to analyse the skin cancer. Mastrolonardo et al. introduced the new technique of the variogram and of fractal analysis extended to the whole regions of interest of skin in order to obtain parameters able to identify the malignant lesion [14]. In another work Hall calculated fractal dimensions to represent border irregularity for early detection of melanoma [15]. In a similar work Piantanelli et al. investigated the fractal properties of skin pigmented lesion boundaries [16]. Ng and Coldman focused on using fractal concept in measuring the fuzziness of a mole. In order to overcome the problem of separation of a mole from its surrounding skin in application of variation method and the correlation method, they employed two different methods which manipulated the intensities around the border of a mole. The first one calculated the size of the intensity surface area at different scales and the second method used the average absolute intensity difference of pixel pairs to obtain normalized fractional feature vectors [17]. See also [18].

In spite of all of these works, no work has been reported which analyses the complexity and correlation of damaged DNA through analysis of DNA walks. In this paper we use the concept of fractal dimension and the Hurst exponent in order to analyse the DNA sequences. In order to do this task we illustrate DNA walk as a random walk and by introducing the fractal dimension and Hurst exponent we compute these parameters for DNA walks which extracted from DNA sequences of healthy subjects and patients with skin cancer. The complexity and correlation of patients' DNA walk are discussed in details.

## RESULTS

In this section we compute the Hurst exponent and fractal dimension for DNA walks in case of healthy subjects and subjects with skin cancer, and then compare the results for diagnosis of skin cancer.

**Figure 1:**
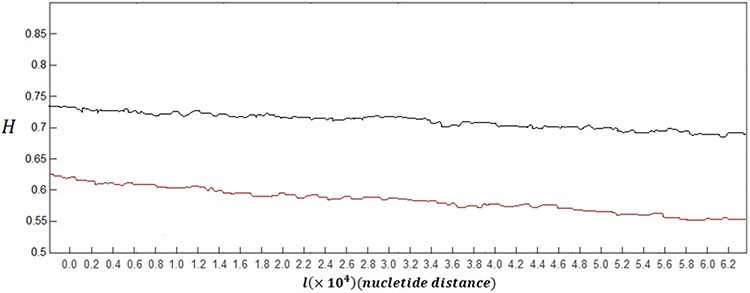
Grand average of the Hurst exponent plots for DNA walks of all healthy subjects (black curve) versus grand average of the Hurst exponent plots for damaged DNA walks of all subjects with skin cancer (red curve)

In order to make a clear comparison, the grand average of the Hurst exponent plots for all of 60 healthy subjects versus the grand average for all of 60 subjects with skin cancer is shown in Figure 1.

As it can be seen in this figure, the overall behaviour of the Hurst exponent variations in case of healthy subjects and also subjects with skin cancer is decreasing behaviour as its value tend to *H* = 0.5. This behaviour stands for the fact that memory of DNA walk is decreasing in the genome. But as it is clear, in case of damaged DNA, variations of the Hurst exponent show steeper behaviour than variations belong to normal DNA walks.

The small upward deflections seen in both curves stand for the small increases in the genome's memory. It is clear that by decreasing the value of *H* (getting closer to 0.5) and accordingly the memory of genome, the predictability of DNA walk is decreasing. But in case of skin cancer DNA walk, the memory and predictability of DNA walks is decreasing faster than normal DNA walks which stands for the fact that the damaged DNA is less able to store information and increases its memory.

Another difference between two curves in Figure 1 can be seen in the values of the Hurst exponent, where in average the Hurst exponent in case of damaged cells has smaller values that are closer to *H* = 0.5 compared to normal DNA. This stands for the fact that there is less correlation in the damaged DNA walk compared to normal DNA walk. The averaged value of the Hurst exponent variations for subjects with skin cancer was computed as 0.579 which is smaller than the computed value for healthy subjects which is 0.715.

**Figure 2:**
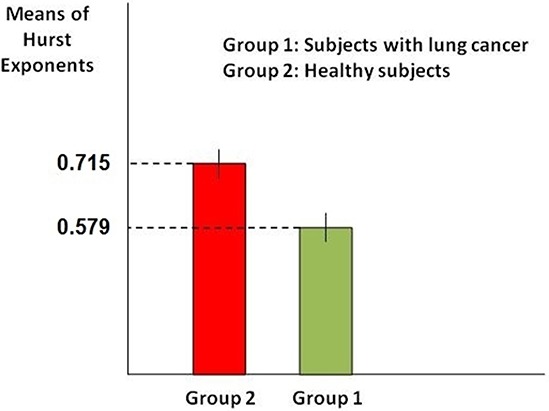
Comparison of confidence interval for means of the Hurst exponent As it is clear in the figure, confidence intervals in case of healthy subjects (red bar) with the variation 0.7138 ≤ *X* ≤ 0.7162 and subjects with skin cancer (green bar) with the variation 0.5775 ≤ *X* ≤ 0.5805 don't overlap, which means they are necessarily significantly different. So this result stands for the significant difference between the Hurst exponents values in case of two groups of subjects.

In order to compare mean of the Hurst exponent values in case of each sample we compute 95% confidence intervals in case of healthy subjects and subjects with skin cancer and then determine whether the intervals overlap. As it is known when 95% confidence intervals for the means of two independent populations don't overlap, there will indeed be a statistically significant difference between the means (at the 0.05 level of significance). Figure 2 shows the computed confidence intervals.

**Figure 3:**
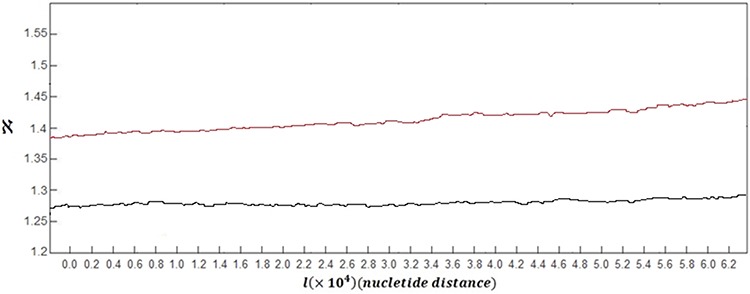
Grand average of the spectra of fractal dimension plots for DNA walks for all of healthy subjects (black curve) versus grand average of the spectra of fractal dimension plots for damaged DNA walks for all of subjects with skin cancer (red curve)

In order to analyse the complexity of DNA walk in case of normal and damaged DNA walks, the fractal dimension spectra of DNA walks are discussed here. The grand average of the spectra of fractal dimension plots for all of 60 healthy subjects versus the grand average for all of 60 subjects with skin cancer is shown in Figure 3.

As it can be seen in this figure, the overall behaviour of the Fractal dimension variations in case of healthy subjects and subjects with skin cancer is increasing behaviour. This behaviour stands for the fact that complexity of DNA walk is increasing in the genome. But as it is clear, in case of damaged DNA, variations of the fractal dimension show steeper behaviour than variations belong to normal DNA walks.

The small downward deflections seen in both curves stand for the small decreases in complexity of DNA because of small increases in the genome's memory. By increasing the fractal dimension's value, the predictability of DNA walk is decreasing as the DNA is getting more complex. But in case of damaged cells, the complexity of DNA walks is increasing faster than normal DNA walks.

Another difference between two curves can be seen in the values of the fractal dimension, where in case of damaged DNA the fractal dimension has bigger values compared to normal DNA and this stands for the fact that the damaged DNA walk is more complex compared to normal DNA walk. The averaged value of the fractal dimension variations for 60 subjects with skin cancer was computed as 1.417 which is bigger than the computed value for healthy subjects which is 1.278.

**Figure 4:**
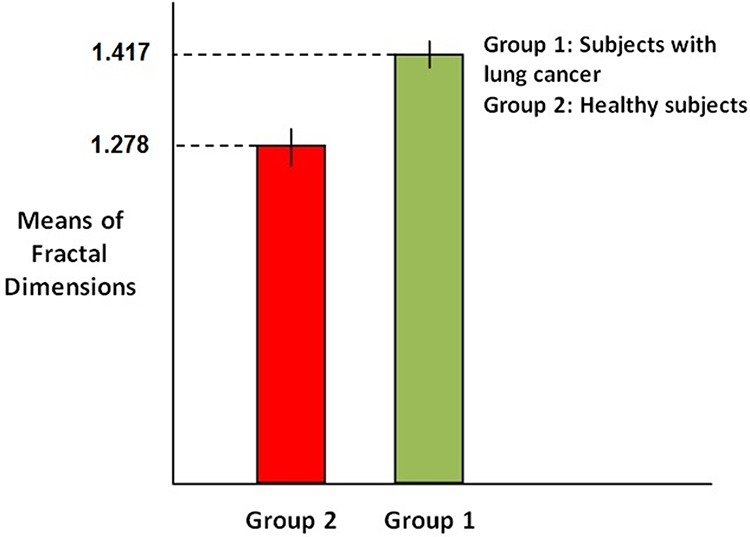
Comparison of confidence interval for means of Fractal dimensions

In order to compare the mean of fractal dimension values in case of each sample we compute 95% confidence intervals in case healthy subjects and subjects with skin cancer and then determine whether the intervals overlap. Figure 4 shows the computed confidence intervals.

As it is clear in the Figure, confidence intervals in case of healthy subjects (red bar) with the variation 1.2768 ≤ *Y* ≤ 1.2792 and subjects with skin cancer (green bar) with the variation 1.4155 ≤ *Y* ≤ 1.4185 don't overlap, which means they are necessarily significantly different. So this result stands for the significant difference between the fractal dimensions values in case of two groups of subjects.

All the analyses which have been done in this research showed that by computing the values of the Hurst exponent and fractal dimension we are able to diagnose the damaged DNA's as they show more complexity and less correlation compared to normal DNA's.

## DISCUSSION

In this paper we worked on diagnosis of skin cancer by analysing the damaged DNA. By defining the Hurst exponent and fractal dimension we talk about correlation and complexity of damaged DNA. The analyses of the Hurst exponent and fractal dimensions plots show that DNA walk have smaller values of the Hurst exponent and bigger values of fractal dimension in case of damaged DNA compared to normal DNA. Also, the Hurst exponent and fractal dimension plots for damaged DNA show steeper behaviour than normal DNA plots. These results stand for the fact that damaged DNA is less predictable and more complex compared to normal DNA. The method used in this research can be applied for analysis and diagnosis of other types of cancer.

## MATERIALS AND METHODS

### DNA and random walk

A DNA sequence is a four-letter (A, C, G, T) text where A, C, G and T stand for the bases adenine, cytosine, guanine and thymine respectively. An example of DNA sequence is

…GTGATAGGGTCTCACTCTGT…

This sequence in letter can be converted to a quaternary number sequence by changing T into 0, A into 1, C into 2, and G into 3, such as

…30310133302021202030….

This genomic sequence is what is contained in the whole set of chromosomes in the nucleus of a single cell. It is a remarkable phenomenon that DNA sequence contained in a cell dictates development of a complete, mature organism from one single cell. Scientists have attempted to decipher the structure and meaning of DNA sequences; however, consensus has not been reached and opinions are diverged.

Various mathematical methods have been applied to investigate the nature of DNA sequences. The chaos game representation of DNA sequences has been reported to produce a unique pattern consistently over different parts of the genome of an organism. From the image generated from the chaos game, characteristics of a DNA sequence can be studied, such as finding association between two letters.

A popular method to graphically portray the genetic information stored in DNA sequences is to use the so-called “DNA walk” representation [19]. DNA Walk is a vectorial representation of DNA sequences transformed into a planar trajectory. It consists first in converting the DNA text into a binary sequence by coding at a given nucleotide positions and at other positions, and then in defining the graph of the DNA walk by the cumulative variables.

A prevalent method for DNA analysis is related to random walk or Brownian motion which led to the discovery of long-range correlation in DNA sequences.

The motion of Brownian particle consists of steps of movement in a characteristic length in a random direction; thus, it's also called a random walk. Suppose the particle moves on the x-axis by jumping +*ξ* or −*ξ* every *τ* seconds, then its movement can be plotted as time proceeds. Likewise, DNA sequence can be plotted in a form of time-series, but the x-axis represents an array of DNA sequence instead of time [19]. This way, the profile of letters can be preserved along the sequence.

DNA sequence can be defined by two of six possible combinations which are purines (A+G), pyrimidines (C+T), Imino (A+C), Keto (G+T), Weak (A+T) and Strong (G+C). Combination of pyrimidine tract with purines tract is long known for analysis of DNA [20]. In this research we chose this combination as it helps for better detection of the long dependence property in DNA sequences (look at [19]).

**Figure 5:**
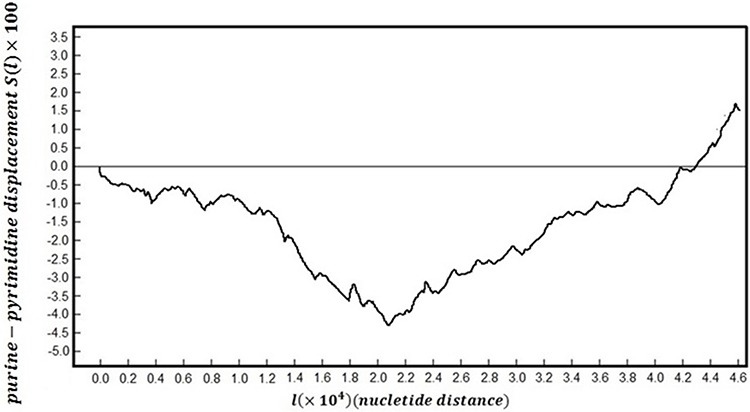
The DNA walk plot

Figure 5 shows map of whole genomic DNA sequences following purine-pyrimidine binary rule: change purines (A/G) to −1 and pyrimidines (C/T) to +1. This creates a one dimensional ‘DNA walk’ along the genome.

In the next section by introducing the Hurst exponent we discuss about the correlation of the random walks and in special case the DNA walks.

### The Hurst exponent and type of motion

In order to analyse the behaviour of a DNA walk, the direction of fluctuation (deflection) from one point to the next point and in a bigger view the correlation of walk should be considered. This behaviour can be studied by computing a time varying parameter, called the Hurst exponent. The Hurst exponent is an indicator of the long term memory of the process and thus, it is the measure of the predictability of the DNA walk.

The Hurst exponent can have any value between 0 and 1, where the value that it gains in each moment determines the behaviour of the next deflection in the signal.

The ‘DNA walker’ moves either up or down at every base pair according to the binary map of the DNA sequence. If there is no long-range correlation, the walk is a realization of a Brownian motion. Otherwise, we observe a ‘walker’ with long-term memory and thus a Fractional Brownian motion. Those two processes can be characterized by different values of the Hurst exponent (H). *H* = 0.5 for Brownian motion and 0 < *H* < 1 · (*H* ≠ 0.5) for Fractional Brownian motion. In case of Brownian motion, *H* = 0.5, the process is considered to be truly random (e.g., Brownian motion). It means that there is absolutely no correlation between any values of the process and it is hard to predict the future of process. The analysis of the Hurst exponent for fractional Brownian motion can be categorized in two ranges. Firstly, if the Hurst exponent has *a* value between 0 and 0.5, it means that the process is anti-persistent i.e. the trend of the process at the next instant will be opposite to the trend in the previous instant. Secondly, *a* value of H between 0.5 and 1 means that the process is persistent i.e., the trend of the process at the next instant will be the same as the trend in the previous instant.

In this research we compute the value of the Hurst exponent for damaged DNA walk and compare its values with the normal DNA walk. This comparison helps us in to find out about the correlation and predictability of damaged versus normal DNA walk.

There are different methods which have been developed to estimate the value of H. Rescaled Range Analysis (R/S) and DFA are two mostly used methods of the Hurst exponent estimation. By the initial analysis of the computed Hurst exponent of DNA walks we found out that even R/S method shows higher values of the Hurst exponent than DFA, the standard deviations are lower for R/S so that the confidence intervals are narrower and thus in our case R/S method is more precise. Nevertheless, we found out that both methods show similar results which become closer as the DNA sequence becomes longer.

So in this research we employ R/S analysis method for computing the Hurst exponent which is described in the next section using a sample.

### Computation of the Hurst exponent using R/S analysis

R/S analysis is described in many literatures as a famous method for calculating the Hurst exponent of time series. Applying this concept to the DNA sequence, the Hurst exponent can be calculated for DNA walk. The same principle which is applied in case of time series also can be applied to DNA sequence. The calculations are explained here through a sample. Considering:

$$X\left(s,l\right)=\sum_{u=1}^{s}\{\xi \left(u\right)-{\langle \xi \rangle }_{l}\}$$ (2)

where *s* is a letter on the sequence of *l* letters long, and

$${\langle \xi \rangle }_{l}=\frac{1}{l}\sum_{s=1}^{l}\xi \left(s\right)$$ (3)

Calculated from table 1, the sum of movements for the entire sequence of length *l* would be

$$\sum_{s=1}^{l}\xi \left(s\right)=-9385-\;6285+\;9300+6122\;=\;-248$$ (4)

So,

$${\langle \xi \rangle }_{l}=\frac{1}{l}\sum_{s=1}^{l}\xi \left(s\right)=\frac{248}{31092}=0.0079763\approx 0$$ (5)

Thus,

$$X\left(s,l\right)=\sum_{u=1}^{s}\{\xi \left(u\right)-{\langle \xi \rangle }_{l}\}\approx \sum_{u=1}^{s}\left\{\xi \left(u\right)-0\right\}=\sum_{u=1}^{s}\left\{\xi \left(u\right)\right\}$$ (6)

Considering adequate letter conversions,

R(l)=maxX(s,l)−minX(s,l) (7)

$$S={\left[\frac{1}{l}\sum_{s=1}^{l}{\left\{\xi \left(s\right)-{\langle \xi \rangle }_{l}\right\}}^{2}\right]}^{1/2}$$ (8)

From (6),

$$R\left(l\right)=\max\sum_{u=1}^{s}\xi \left(u\right)-\min\sum_{u=1}^{s}\xi \left(u\right)$$ (9)

Since $\sum_{u=1}^{s}\xi \left(u\right)$ is the position of a letter s along the y-axis, *R*(*l*) is equivalent to the difference between the maximum point and the minimum point on the DNA walk; thus, from Figure 5,

R(l)= 175−(−440) = 615 (10)

From (5) and (8)

$$S={\left[\frac{1}{l}\sum_{s=1}^{l}{\left\{\xi \left(s\right)-0\right\}}^{2}\right]}^{1/2}={\left[\frac{1}{l}\sum_{s=1}^{l}{\left(\xi \left(s\right)\right)}^{2}\right]}^{1/2}\approx 1$$ (11)

So

$$R/S\approx R\left(l\right)={\left(\frac{l}{2}\right)}^{H}$$ (12)

Consequently,

$$H=\frac{\log R(l)}{\log(\frac{l}{2})}=\frac{\log615}{\log(\frac{31092}{2})}\approx 0.663$$ (13)

Based on the last discussion the value of *H* suggests that there exists good persistence in the DNA walk as it is between 0.5 and 1.

**Table 1:** Probability, number of occurrence (bp), and movement of each nucleotide

Nucleotide	Probability	Number of occurrence (bp)	Movement
A	0.3018	9385	−1
G	0.2021	6285	−1
T	0.2991	9300	+1
C	0.1968	6122	+1
Total	1	31092	

In order to make a comparison, some of published values of *H* for DNA sequences and the value computed in Equation (13) are brought in Table 2.

**Table 2:** Some published values of *H* for DNA sequences

Sequence	*H*	Reference
human beta-cardiac myosin heavy chain gene	0.67	[21]
human beta globin purine-pyrimidine representation	0.708	[22]
synthetic model sequence	0.655	[22]
DNA genetic sequences	0.663	Governed in Equation (13)

As it can be seen in Table 2, there are good correlations between our governed value in Equation (13) and other researchers' computed values in all cases for normal DNA.

Estimation of the Hurst exponent by R/S analysis is more laborious than this naive estimation. In this research R/S value is calculated for *l*, *l*/2, *l*/4,…, and *l*/2^n^, and for each division of *l*, average R/S is calculated again. Then a linear regression line is obtained from plotting log(R/S) versus log*l*. Then, the slope of the linear graph is the estimated Hurst exponent. In this research we calculate the Hurst exponent in different segments of DNA walk and report a signal-shaped plot for it not only an average value. Using this method we are able to talk about the memory and predictability in the DNA walk.

### Spectra of fractal dimension

In this section we explain the fractal dimension as a measure of complexity of the fractals and accordingly DNA walk.

The concept of fractal dimension is based on the concept of generalized entropy of a probability distribution, introduced by Renyi [23]. In case of a DNA walk with *ξ*_max_ and *ξ*_min_, where the total range of the value is divided into *N* bin:

$$N=\frac{\xi_{max}-\xi_{min}}{\delta \xi }$$ (14)

The probability that the value falls into the *i*-th bin of size *δξ* is computed as:

$$w_{i}=\lim_{N\to \infty }\frac{N_{i}}{N}$$ (15)

where *N*_*i*_ equals the number of items the value falls into the *i*-th bin. On the other hand, in case of a DNA walk:

$$w_{i}=\lim_{l\to \infty }\frac{s_{i}}{l}$$ (16)

where *s_i_* is letter in the *i*-th bin in the entire sequence of length *l*.

Starting with the letter of order *q* of the probability *w_i_*, the Renyi entropy is:

$$E_{q}=\frac{1}{1-q}log_{2}\sum_{i=1}^{N}w_{i}^{q}$$ (17)

Note that for *q* → 1:

$$E_{1}=-\sum_{i=1}^{N}w_{i}logw_{i}$$ (18)

The generalized fractal dimensions of a given DNA walk with the known probability distribution are defined as:

$${ℵ}_{q}=\lim_{\delta \xi \to 0}\frac{1}{q-1}\frac{\log\sum_{i=1}^{N}w_{i}^{q}}{\log\delta \xi }$$ (19)

where the parameter *q* ranges from − ∞ to + ∞. Note that for a self-similar (simple) series with equal probabilities *w_i_* = 1/*N*, equation (19) yields ℵ_*q*_ = ℵ_0_ for all values of *q*. Also, note that for a constant value, all probabilities except one become equal to zero, whereas the remaining probability value equals unity.

For a given DNA walk, the function ℵ_*q*_, corresponding to the probability distribution of walk, is called the fractal spectrum. Indeed, a larger value of the fractal dimension for a given DNA walk corresponds to the presence of more pronounced fluctuations (sharper fluctuations, less expected values of the DNA walk) than in the DNA walks for which the value of fractal dimension of the same order is less. Furthermore, DNA walks with a wider range of fractal dimensions can be termed more fractal than DNA walks whose range of fractal dimensions is narrower, so that DNA walks with the zero range are self-similar (simple) fractals.

Now, if the unexpectedness of an event is defined as the inverse of the probability of this event, then steeper spectra correspond to the series in which unexpected values are more dominant, whereas flatter spectra represent those series in which less unexpectedness occurs [9].

### Data collection

In order to do the analyses, the sequences of interest were collected from 60 patients with skin cancer (30 male and 30 female) and 60 subjects in complete healthy conditions (30 male and 30 female) that all of them are 35 ± 3 years old. It is noteworthy that patients are in early steps of melanoma cancer. Patients didn't receive any treatment (chemotherapy, etc.) before their recruitment. Before doing the experiments each subject was interviewed by a physician to describe the nature of experiments and then informed consent was obtained from them. It is noteworthy that all procedures were approved by the Internal Review Board of the University. Identity of all subjects remains confidential.

In this research sample were taken from subjects' skin. Samples were homogenized in the TissueLyser II from Qiagen for 1 min at 28 Hz, centrifuged for 60 s at 3000 × g and incubated at 56°C for 30 min. After centrifugation at 6000 × g for 20 min the clarified samples were transferred to MACHEREY-NAGEL 8-well strips on the Microlab^®^ STAR and further processed according to the MACHEREY-NAGEL NucleoSpin^®^ 8 Plant protocol. The MACHEREY-NAGEL NucleoSpin 8 Plant kit was used for the extraction of genomic DNA based on vacuum filtration. The extraction yields high quality DNA suitable for further analyses.

### Data analysis

In order to do the analyses a program was written in MATLAB to generate the DNA walk for the sequences. This program maps the whole genomic DNA sequences using purine-pyrimidine binary rule by changing purines (A/G) to −1 and pyrimidines (C/T) to +1. This procedure creates the DNA walk along genome. After this being established the DNA walk is analysed by computing the Hurst exponent and Fractal dimension.

## References

[1] Cakir BÖ, Adamson P, Cingi C (2008). Epidemiology and economic burden of non-melanoma skin cancer. Facial Plast Surg Clin North Am.

[2] Rajpar S, Marsden J (2008). ABC of skin cancer.

[3] Hendi A, Martinez JC (2011). Atlas of Skin Cancers: Practical Guide to Diagnosis and Treatment.

[4] Segura S, Puig S, Carrera C, Palou J, Malvehy J (2009). Development of a two-step method for the diagnosis of melanoma by reflectance confocal microscopy. The American Academy of Dermatology Journal.

[5] Stoecker WV, Li WW, Moss RH (1992). Automatic detection of asymmetry in skin tumors. Computerized Medical Imaging and Graphics.

[6] Celebi ME, Iyatomi H, Schaefer G, Stoecker WV (2009). Lesion border detection in dermoscopy images. Computerized Medical Imaging and Graphics.

[7] Korotkov K, Garcia R (2012). Computerized analysis of pigmented skin lesions: A Review. Artificial Intelligence in Medicine.

[8] Celebi ME, Schaefer G (2012). Color medical image analysis. Springer. lecture notes in computational vision and biomechanics.

[9] Scharcanski J, Celebi ME (2013). Computer vision techniques for the diagnosis of skin cancer. Springer, Series in bioengineering.

[10] Flores-Sahagun JH, Vargasa JVC, Mulinari-Brenner FA (2011). Analysis and diagnosis of basal cell carcinoma (BCC) via infrared imaging. Infrared Physics & Technology.

[11] Poljak-Blazi M, Kolaric D, Jaganjac M, Zarkovic K, Skala K, Zarkovic N (2009). Specific thermographic changes during Walker 256 carcinoma development: Differential infrared imaging of tumour, inflammation and haematoma. Cancer Detection and Prevention.

[12] Cholewka A, Stanek A, Kwiatek S, Sieroń A, Drzazga Z (2013). Does the temperature gradient correlate with the photodynamic diagnosis parameter numerical colour value (NCV)?. Photodiagnosis and Photodynamic Therapy.

[13] Kulish VV (2010). Partial differential equations.

[14] Mastrolonardo M, Conte E, Zbilut JP (2006). A fractal analysis of skin pigmented lesions using the novel tool of the variogram technique. Chaos, Solitons & Fractals.

[15] Hall PN, Claridge E, Smith JDM (1995). Computer screening for early detection of melanoma-is there a future?. British Journal of Dermatology.

[16] Piantanelli A, Maponi P, Scalise L, Serresi S, Cialabrini A, Basso A (2005). Fractal characterisation of boundary irregularity in skin pigmented lesions. Medical and Biological Engineering and Computing.

[17] Ng V, Coldman A (1993). Diagnosis of Melanoma with Fractal Dimensions. Proceedings of the IEEE Region 10 Conference on Computer, Communication. Control and Power Engineering.

[18] Ng V, Lee T (1996). Measuring Border Irregularities of Skin Lesions Using Fractal Dimensions. Proc. SPIE 2898. Electronic Imaging and Multimedia Systems.

[19] Peng CK, Buldyrev S, Goldberger A, Havlin S, Sciortino F, Simons M, Stanley HE (1992). Long-range correlations in nucleotide sequences. Nature.

[20] Yagil G (2004). The over-representation of binary DNA tracts in seven sequenced chromosomes. BMC Genomics.

[21] Peng CK, Buldyrev SV, Goldberger AL, Havlin S, Sciortino F, Simons M, Stanley HE (1992). Fractal landscape analysis of DNA walks. Physicia A.

[22] Borovik AS, Grosberg AY, Kamenetskii F (1994). Fractality of DNA texts. J. Biomol Struct.

[23] Renyi A (1906). On a new axiomatic theory of probability. Acta Mathematica Hungarica.

